# Multiparametric assessment of mitochondrial respiratory inhibition in HepG2 and RPTEC/TERT1 cells using a panel of mitochondrial targeting agrochemicals

**DOI:** 10.1007/s00204-020-02792-5

**Published:** 2020-07-18

**Authors:** Wanda van der Stel, Giada Carta, Julie Eakins, Salihanur Darici, Johannes Delp, Anna Forsby, Susanne Hougaard Bennekou, Iain Gardner, Marcel Leist, Erik H. J. Danen, Paul Walker, Bob van de Water, Paul Jennings

**Affiliations:** 1 Division of Drug Discovery and Safety, Leiden Academic Centre of Drug Research, Leiden University, Einsteinweg 55, 2333 CC Leiden, The Netherlands; 2 Division of Molecular and Computational Toxicology, Department of Chemistry and Pharmaceutical Sciences, AIMMS, Vrije Universiteit Amsterdam, De Boelelaan, 1108, 1081 HZ Amsterdam, The Netherlands; 3 Cyprotex Discovery Ltd, Alderley Park, Macclesfield, Cheshire, UK; 4 University of Konstanz, Constance, Germany; 5 Department of Biochemistry and Biophysics, Stockholm University, Stockholm, Sweden; 6 National Food Institute Technical University of Denmark (DTU), Lyngby, Denmark; 7 Certara UK Limited, Sheffield, UK

**Keywords:** Mitochondria, Seahorse, ETC, ECAR, MMP, RPTEC/TERT1, HepG2

## Abstract

Evidence is mounting for the central role of mitochondrial dysfunction in several pathologies including metabolic diseases, accelerated ageing, neurodegenerative diseases and in certain xenobiotic-induced organ toxicity. Assessing mitochondrial perturbations is not trivial and the outcomes of such investigations are dependent on the cell types used and assays employed. Here we systematically investigated the effect of electron transport chain (ETC) inhibitors on multiple mitochondrial-related parameters in two human cell types, HepG2 and RPTEC/TERT1. Cells were exposed to a broad range of concentrations of 20 ETC-inhibiting agrochemicals and capsaicin, consisting of inhibitors of NADH dehydrogenase (Complex I, CI), succinate dehydrogenase (Complex II, CII) and cytochrome bc1 complex (Complex III, CIII). A battery of tests was utilised, including viability assays, lactate production, mitochondrial membrane potential (MMP) and the Seahorse bioanalyser, which simultaneously measures extracellular acidification rate [ECAR] and oxygen consumption rate [OCR]. CI inhibitors caused a potent decrease in OCR, decreased mitochondrial membrane potential, increased ECAR and increased lactate production in both cell types. Twenty-fourhour exposure to CI inhibitors decreased viability of RPTEC/TERT1 cells and 3D spheroid-cultured HepG2 cells in the presence of glucose. CI inhibitors decreased 2D HepG2 viability only in the absence of glucose. CII inhibitors had no notable effects in intact cells up to 10 µM. CIII inhibitors had similar effects to the CI inhibitors. Antimycin A was the most potent CIII inhibitor, with activity in the nanomolar range. The proposed CIII inhibitor cyazofamid demonstrated a mitochondrial uncoupling signal in both cell types. The study presents a comprehensive example of a mitochondrial assessment workflow and establishes measurable key events of ETC inhibition.

## Introduction

There is accumulating evidence that chemical-induced organ toxicity involves disruption of mitochondrial function more frequently than previously considered (Dykens and Will 2007; Will et al. 2019; Dreier et al. 2019). Mitochondrial perturbations can have major effects on tissues and organs due to their key role in fatty acid metabolism, energy production and generation of reactive oxygen species (ROS). There are several mechanisms of direct mitochondrial perturbation including electron transport chain (ETC) inhibition, mitochondrial DNA damage, ROS, cardiolipin binding, Krebs cycle inhibition, disturbances of fatty acid shuttling, beta oxidation inhibition and protonphoretic (uncoupling) activity (Boelsterli 2003). The subsequent dysfunction of these organelles can have several adverse effects, which is both dependent on the target tissue’s reliance on mitochondrial function and the type of mitochondrial perturbation.

Various chemical classes may pose human liability for mitochondrial toxicity. Several drugs have been withdrawn from the market due to organ toxicity, which has subsequently been proven or has strong evidence supporting a central role for mitochondrial perturbation (Nadanaciva et al. 2007; Dykens and Will 2008; Longo et al. 2016; Eakins et al. 2016; Grünig et al. 2017). Compounds that fail late in clinical trials, or those that are withdrawn from the market are costly in terms of financial and time resources, but also on patient’s health. The assessment of the potential of drug candidates to perturb mitochondria should be a fundamental parameter in the early stage of drug development to prevent later, often devastating, adverse drug reactions in patients. Furthermore, the agrochemical industry has harnessed a broad range of effective pesticides and fungicides that act via targeting individual complexes of the ETC. Selective inhibition of CI and CIII by model mitochondrial toxins has been associated with adverse responses in pre-clinical species, including neurological defects (Cannon et al. 2009). Therefore, a thorough assessment of mitochondrial toxicity could also provide important input for risk assessment in the case of industrial chemicals and environmental pollutants. While various divergent assays have been established to assess mitochondrial perturbations, there is no current consensus on the most appropriate assays to use, which combinations nor on the most appropriate cell types.

In this study, we aimed to systematically assess the applicability of several assays which could eventually form the basis of a consensus mitochondrial toxicity testing platform. To this end, we used two human cell lines, the renal RPTEC/TERT1 and the hepatic HepG2 cells. RPTEC/TERT1 are a non-cancerous human telomerase immortalised cell line that exhibit a differentiated oxidative phenotype when differentiated via contact inhibition (Aschauer et al. 2013). The HepG2 cell line under standard 2D conditions exhibit a highly proliferative phenotype, but can be further differentiated under 3D spheroid conditions (Ramaiahgari et al. 2014). We chose a panel of 20 agrochemicals which have been harnessed for the selective inhibition of ETC, consisting of inhibitors of NADH dehydrogenase (CI), succinate dehydrogenase (CII) and cytochrome bc1 complex (CIII). Capsaicin was also included due to its proposed CI activity (Satoh et al. 1996). A battery of assays was utilised including assays monitoring viability, lactate production, mitochondrial membrane potential, and the simultaneous quantification of extracellular acidification and cellular oxygen consumption. While this study focuses on ETC inhibition, the combination of these assays has the potential to measure the majority, if not all, mitochondrial perturbations.

## Materials and methods

### Chemicals

**Fig. 1:**
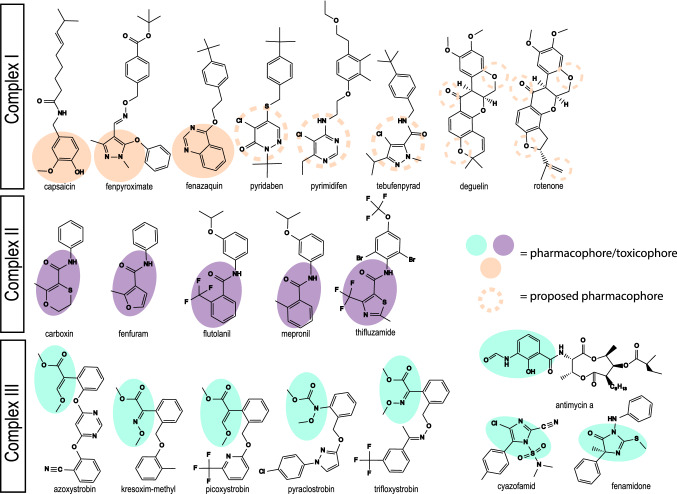
Chemical structures of selected ETC complex I, II, and III inhibitors. Molecular structures of all assessed mitochondrial complex inhibitors organised based on their MoA (Table 1). The filled orange, purple and green areas highlight the region involved in the molecular recognition needed to perform the molecule´s activity for CI, CII and CIII inhibitors respectively. The dashed orange circles indicate the proposed regions of the pharmacophores for the remaining complex I inhibitors (color figure online)

All tested compounds were purchased from Merck at one site (JRC, Ispra, Italy) and distributed to the testing laboratories. The catalogue numbers are Capsaicin (Cat. No. M2028), Deguelin (D0817), Fenazaquin (31635), Fenpyroximate (31684), Pyridaben (46047), Pyrimidifen (35999), Rotenone (R8875), Tebufenpyrad (46438), Carboxin (45371), Fenfuram (45486), Flutolanil (N12004), Mepronil (33361), Thifluzamide (49792), Antimycin A (A8674), Azoxystrobin (3167), Cyazofamid (33874), Fenamidone (33965), Kresoxim-methyl (37899), Picoxystrobin (33568), Pyraclostrobin (33696), and Trifloxystrobin (46477). The compounds are listed by class in Table 1 and structural information is provided in Fig. 1. Stock solutions between 10 and 100 mM were created in dimethyl sulfoxide (DMSO) and stored at − 20 °C or − 80 °C until use. Treatment solutions were prepared freshly from DMSO stocks for each experiment and the final concentration of DMSO in the systems was 0.1% (v/v). For repeated administration, the culture medium was removed and the new medium with the compound was added every 24 h.

**Table 1:** General properties of selected ETC complex I, II, and III inhibitors

Panel compounds	ETC complex inhibited	CAS number	Molecular weight (g/mol)	ClogP	Application	Chemical group	Inhibitor type	Putative binding site	References
Capsaicin*	I	404-86-4	305.20	3.64	Topical analgesic, pepper spray agent	Phenol	Type C	Quinone binding pocket	Degli Esposti and Ghelli (1994), Tocilescu et al. (2010)
Deguelin*	I	522-17-8	394.14	4.26	Insecticide	Flavonoid		Quinone binding pocket	Degli Esposti and Ghelli (1994)
Fenazaquin	I	120928-09-8	306.17	5.51	Insecticide/acaricide	Quinazoline	Type A	Quinone binding pocket	Wood et al. (1996), Rgen et al. (1999)
Fenpyroximate*	I	134098-61-6	421.20	5.01	Acaricide	Pyrazoles	Type A	Quinone binding pocket	Rgen et al. (1999), Ino et al. (2003), Tocilescu et al. (2010)
Pyridaben	I	96489-71-3	364.14	5.24	Insecticide/acaricide	Pyrimidine		Quinone binding pocket	Schuler et al. (1999)
Pyrimidifen*	I	105779-78-0	377.19	5.03	Insecticide/acaricide	Pyrimidine	Type A	Quinone binding pocket	(Lümmen 1998; Rgen et al. 1999)
Rotenone*	I	83-79-4	394.14	4.10	Insecticide	Flavonoid	Type B	Quinone binding pocket	Degli Esposti and Ghelli (1994), Tocilescu et al. (2010)
Tebufenpyrad*	I	119168-77-3	333.16	4.93	Insecticide/acaricide	Pyrazoles		Quinone binding pocket	Degli Esposti (1998)
Carboxin*	II	5234-68-4	235.07	2.22	Fungicide	Oxathiin-carboxamides	Qp	Quinone binding site	Horsefield et al. (2006), Huang et al. (2006), Ruprecht et al. (2009), Sierotzki and Scalliet (2013)
Fenfuram	II	24691-80-3	201.08	2.24	Fungicide	Furan- carboxamides	Qp	Quinone binding site	Sierotzki and Scalliet (2013)
Flutolanil	II	66332-96-5	323.11	3.70	Fungicide	Phenyl-benzamides	Qp	Quinone binding site	Sierotzki and Scalliet (2013)
Mepronil*	II	55814-41-0	269.14	3.90	Fungicide	Phenyl-benzamides	Qp	Quinone binding site	Sierotzki and Scalliet (2013), Kluckova et al. (2015)
Thifluzamide*	II	130000-40-7	525.84	5.05	Fungicide	Thiazole-carboxamides	Qp	Quinone binding site	Sierotzki and Scalliet (2013)
Antimycin A*	III	1397-94-0	548.27	4.41	Piscicide		Qi	Q-cycle	Gao et al. (2003), Esser et al. (2004, 2014), Zhao et al. (2010)
Azoxystrobin*	III	131860-33-8	403.12	2.50	Fungicide	Methoxy-acrylates	Qo, Pm	Q-cycle	Esser et al. (2004, 2014), Zhao et al. (2010)
Cyazofamid*	III	120116-88-3	324.04	3.20	Fungicide	Cyano-imidazole	Qi	Q-cycle	Esser et al. (2014)
Fenamidone	III	161326-34-7	311.11	3.72	Fungicide	Imidazolinones	Qo, Pf	Q-cycle	Esser et al. (2014)
Kresoxim-methyl	III	143390-89-0	313.13	3.40	Fungicide	Oximino-acetates	Qo, Pm	Q-cycle	Esser et al. (2004, 2014), Zhao et al. (2010)
Picoxystrobin*	III	117428-22-5	367.10	3.81	Fungicide	Methoxy-acrylates	Qo, Pm	Q-cycle	Esser et al. (2004, 2014), Zhao et al. (2010)
Pyraclostrobin*	III	175013-18-0	387.10	3.99	Fungicide	Methoxy-carbamates	Qo, Pm	Q-cycle	Esser et al. (2004, 2014), Zhao et al. (2010)
Trifloxystrobin	III	141517-21-7	408.13	4.50	Fungicide	Oximino-acetates	Qo, Pm	Q-cycle	Esser et al. (2004, 2014), Zhao et al. (2010)

*Refers to the subset of panel compounds in this study. **CI** (NADH:ubiquinone oxidoreductase), **CII** (succinate dehydrogenase), **CIII** (cytochrome *bc*_1_ complex). **ClogP**: lipophilicity as reported by Delp et al. (2019). Chemical group from FRAC Code List © 2019. Inhibitor: type A—quinone antagonist, type B—semiquinone antagonist, type C—quinol antagonist, Qo—outside quinol oxidation pocket, Qi inside quinone reduction pocket, Pf-Qo sub-type I, Pm-Qo sub-type II

### Cell culture

The human renal proximal tubule-derived cell line RPTEC/TERT1, is a non-cancerous cell line which was immortalised by introduction of the catalytic unit of human telomerase (hTERT) (Wieser et al. 2008). These cells were obtained under licence from Evercyte GmBH, Vienna Austria. RPTEC/TERT1 grow in a monolayer and after reaching confluence become contact-inhibited, enter cell cycle arrest and differentiate into a transporting epithelium (Aschauer et al. 2013). RPTEC/TERT1 at passage numbers between 72 and 95 were routinely cultured in 10 cm dishes (Sarstedt, 83.3902) at 37 °C in a 5% CO_2_ humidified atmosphere. Cells were fed every second to third day with medium containing 1:1 mixture of Dulbecco’s modified Eagle’s medium (DMEM, no glucose, Invitrogen, 11966) and Ham’s F-12 nutrient mix (Invitrogen, 21765), with a final glucose concentration of 5 mM, supplemented with 2 mM glutamax (Thermofisher, 350500038), 5 µg/L insulin, 5 µg/L transferrin and 5 ng/L sodium selenite (Sigma-Aldrich, I1884), 100 U/mL penicillin and 100 µg/mL streptomycin (Merck, P4333), 10 ng/mL epithelial growth factor (Merck, E9644), 36 ng/mL hydrocortisone (Merck, E9644) and 0.5% foetal bovine serum (Gibco, 10720-106). For experiments, cells were plated in a required format plate, allowed to become contact-inhibited and fed 24 h prior to treatment exposure. For experiments in galactose condition, cells were fed 24 h prior to experiment with culture medium (custom made DMEM/F12, PromoCell) in which 5 mM glucose was replaced with 5 mM galactose (Merck, G5388).

The HepG2 cell line, a human hepatocellular carcinoma, was obtained from ATCC (American Type Culture Collection, Wesel, Germany). HepG2 were cultured in Dulbecco’s modified Eagle’s medium (DMEM; 25 mM glucose, 4 mM l-glutamine, 1 mM sodium pyruvate) (Fisher Scientific, 11504496) supplemented with 10% (v/v) foetal bovine serum (Fisher Scientific, S181L-500), 25 U/mL penicillin and 25 μg/mL streptomycin (Fisher Scientific, 15070-063). Cells were maintained at 37 °C in a 5% CO_2_ humidified atmosphere, fed every 2 to 3 days and passaged at approximately 80% confluence. For 2D HepG2 culture, the cells plated 48 h before exposures in 384 black µclear plates (Greiner Bio-One, 781 091) with a density of 10,000 cells/well. For galactose experiments, the medium was refreshed 24 h before exposure with galactose-containing medium. Galactose medium consists of glucose-free DMEM (Fisher Scientific, 11966-025), 10 mM galactose (Merck, G5388-100G), 1 mM sodium pyruvate (Sigma, P2256-100 g), 10% (v/v) dialysed foetal bovine serum (GE healthcare, 26400-044), 25 U/mL penicillin and 25 μg/mL streptomycin (Fisher Scientific, 15070-063). The protocol for HepG2 3D culture was described previously (Ramaiahgari et al. 2014). In short, Matrigel™ (BD biosciences, 354230) was diluted with ice-cold PBS to 5 mg/mL. 10 μL was used to coat a 384-well Screenstar plate (Greiner, 781866). Cells were seeded at 1000 cells per well in DMEM/Hams F12 (Thermo Fisher, 21041033) supplemented with 10% FBS, 25 U/mL penicillin and 25 μg/mL streptomycin. Culture medium was refreshed every 3–4 days and the culture was maintained for 21 days prior to dosing.

### Resazurin assay

The resazurin reduction assay was conducted as previously described (Jennings et al. 2007). Briefly, an 880 µM resazurin stock solution (20×) was generated by dissolving 0.011 g resazurin (Merck, R7017), in 0.1 N NaOH and bringing to 50 mL in phosphate buffer and adjusting pH to 7.8. After exposure to compounds, supernatant was replaced with 44 µM resazurin stock in cell culture medium and incubated for 1.5 h to 2 h, at 37 °C in a 5% CO_2_ humidified atmosphere. The conversion of resazurin to fluorescent resorufin was measured in a plate reader at excitation/emission 540/590 nm.

### Mitostress assay in intact cells with Seahorse XFe96 Bioanalyzer

The Seahorse bioanalyzer simultaneously measures cellular oxygen consumption rates (OCR) and extracellular acidification rates (ECAR). The mitostress assay utilises a sequential addition of modulators of the oxidative phosphorylation to assess key parameters of mitochondrial function. Subsequent injection of oligomycin (Merck, O4876), FCCP (Merck, C2920) and a mixture of rotenone (Merck, R8875) and antimycin A (Merck, A8674) provide information on ATP production, maximal respiration rates and non-mitochondrial respiration, respectively. RPTEC/TERT1 cells were seeded onto Seahorse XF96 V3 PS Cell Culture Microplates (Agilent, 101085-004) at the density of 25,000 cells/well and allowed to differentiate for a minimum of 2 weeks before assay. It is worth noting here that RPTEC/TERT1 require longer differentiation time in the Seahorse plates, potentially due to sub-optimum gas exchange in the Seahorse culture plates. HepG2 cells were cultured at the density of 15,000 cells/well on collagen-coated (2.5 µg/µL collagen IV, Merck, C7521) Seahorse cell culture microplates two days prior to analysis.

The mitostress test was performed as described (Eakins et al. 2016; Tilmant et al. 2018). Immediately before the assay, cell culture medium was replaced with 180 µL of Seahorse XF Base Medium without phenol red (Agilent, 1003335-100), supplemented with 10 mM d-glucose (Merck, G7021), 5 mM HEPES (Merck, H4034), 2 mM sodium pyruvate (Merck, P5281) and 1 mM l-glutamine (Merck, G8540). Cells were allowed to equilibrate for 45 min in a non-CO_2_ 37 °C incubator. Agilent XFe96 sensor cartridge was hydrated 24 h prior to experiments with Seahorse XF Calibrant (Agilent, 100840-000) and both placed into the Seahorse bioanalyser for assay. Compounds were injected sequentially. OCR was measured five times after test compound injection and three times for all injections. Each measurement consisted of a 3 min mix and subsequent 3 min read cycle. The sequence of injection was as follows: (A) test compound at 8 concentration points (1 in 5 dilutions starting at 10 µM final), (B) oligomycin (2 µM), (C) FCCP (2 µM) and (D) rotenone/antimycin A (0.5 µM each). OCR measurement was normalised to the baseline OCR measurement prior to compound addition. Measurements were performed in triplicate (3 wells) for each independent experiment.

### Mitochondrial complex assay with Seahorse XFe96 Bioanalyser

The mitochondrial complex assay uses real-time OCR measurement with sequential addition of specific complex substrates and/or inhibitors to identify the complex within the ETC that is inhibited. The assay principle explained in detail by Salabei et al. (2014) is to sequentially target specific electron chain complexes. In the first step, cultured cells are permeabilised and provided with CI substrates. The test compound is injected and OCR is measured. In the second step, rotenone and succinate are added which simultaneously block CI while suppling substrates to CII. OCR is measured for the second time. In the third step, Antimycin A together with ascorbate and tetramethyl phenylenediamine (TMPD) is added to simultaneously block CIII and supply CIV. A third and final OCR measurement is conducted. From the patterns of OCR inhibition, one can determine at which part of the ETC the compound is exhibiting its effect (Fig. 4).

HepG2 cells were seeded at 20,000 cells/well on Seahorse XF96 V3 PS cell culture microplates (Agilent, 101085-004). The following day, cells were washed once in mitochondrial assay solution (MAS), containing 220 mM mannitol (Sigma, M9647), 70 mM sucrose (Sigma, S7903), 10 mM potassium phosphate (Sigma, P5655), 5 mM magnesium chloride (Sigma, M8266), 3 mM HEPES (Sigma, H0887) and 1 mM EGTA (Sigma, E4378) with 0.2% fatty acid-free BSA (Sigma, A8806). This solution was replaced with 180 µL MAS supplemented with 10 mM pyruvate (Sigma, 107360), 1 mM malate (Sigma, M0875), 4 mM ADP (Sigma, A5285), 0.2% fatty acid-free bovine serum albumin and 2 nM XF plasma membrane permeabilizer (PMP) (Agilent, 102504-100) and placed immediately into the Seahorse bioanalyser. Each measurement consisted of a thirty second mix and two-minute read cycle. Compounds were injected sequentially and OCR was measured twice. The sequence of injection was as follows: test compound, succinate (10 mM, Merck, S9512) and rotenone (2 µM, Chem Cruz, Sc203242) and TMPD (0.5 mM, Merck, T7394), ascorbate (10 mM, Merck, A5960) and antimycin A (2 µM, Merck, A8674). Complex inhibition was determined using the second OCR measurement after compound/vehicle injection, normalised to the baseline OCR measurements prior to compound addition. ETC inhibition is determined in decreased OCR. CI inhibition is confirmed if the inhibited OCR by injection A can be rescued after injection B. CII inhibition is determined if there is no inhibition after A, but inhibition after B. CIII inhibition is determined if there is inhibition after A and B, with recovery after C. OCR inhibition after injection C indicates effects downstream of CIII.

### Mitochondrial membrane potential (MMP) changes assays with JC-1

JC-1 is a single-excitation dual-emission fluorescence-based assay that allows for ratiometric semiquantitative assessment of mitochondrial membrane potential (Perry et al. 2011). For quantification of changes in MMP cells were pre-loaded, for approximately 1.5 h, with the JC-1 pre-loading solution containing 9 µM JC-1 (Invitrogen, 65-0851-38), 9 µL/mL Pluronic F-127 10% in water (Invitrogen, P6866) and 5 µM Cyclosporine A (CsA) (Merck, 30024), a P-glycoprotein (P-gp) inhibitor, to prevent dye extrusion due to the expression of efflux pumps in RPTEC/TERT1 cell line. After this incubation time, cells were washed and exposed to test compounds dissolved in a JC-1 treatment solution containing 0.5 µM JC-1, 1.5 µL/mL Pluronic F-127 and 1 µM CsA for the desired time. We have previously determined that CsA at up to 5 µM has no adverse effect on RPTEC/TERT1 cells for up to 14 days (Wilmes et al. 2013). Fluorescence was measured in a plate reader at excitation 492 nm and dual emission, 535 nm for monomers and 590 nm for dimers. Results are presented as the dimer/monomer ratio and expressed as percentage of untreated samples.

### Mitochondrial membrane potential plus cell death assay using live confocal imaging

Rhodamine 123 (Rho123) (Merck, R8004) was used in a live confocal imaging setting for the assessment of effects at MMP. Rho123 localises to mitochondria and the decline in fluorescence is proportional to a decrease in MMP (Johnson et al. 1980, 1981). HepG2 were seeded at 10,000 cells/30 μL/well in a 384-wells μCLEAR^®^ black plate (Greiner Bio-One, 781 091). Two days post-seeding, 30 μL complete HepG2 culture medium, containing 200 ng/μL Hoechst 33342 (Life technologies, H1399) and 1 μM Rho123, was added to the medium. After 1 h incubation at 37° C, the medium was removed and 25 μL complete DMEM containing 0.2 μM Rho123 and 100 nM propidium iodide (PI) (Merck, P4170) was added, followed by 25 μL of medium containing 2×, the desired concentration of test compound. The intensity of Hoechst, Rho123 and PI, was monitored using live confocal imaging for 24 h using the 408, 488 and 561 nm laser, respectively. The confocal imaging was performed using a Nikon TiE2000 with perfect focus system and xy-stage (Nikon, Amsterdam, The Netherlands). Quantification of the Hoechst, Rho123 and PI signal intensity and localization were performed using CellProfiler version 2.1.1 (Broad Institute, Cambridge, USA). The nuclear identification based on the Hoechst signal was performed using an internally created segmentation module (Di et al. 2012), followed by a cytoplasmic identification based on a specific distance from these nuclei. The PI-positive nuclei were identified by masking the previous segmented nuclei and the PI signal. More than 10% overlap was considered as PI positive. All CellProfiler analyses were stored as HDF5 files. The combination of an internally developed R script, run in Rstudio (Boston, USA), was used for data extraction including Rho123 signal intensity, fraction PI positive and nuclear count.

### Lactate assay

The colorimetric assay for the lactate detection is based on the conversion of lactate to pyruvate by the lactate dehydrogenase (LDH) enzyme. The process is coupled with the active reduction of the co-factor NAD to NADH. NADH reduces *N*-methylphenazonium methyl sulfate (PMS) to PMSH which reduces *p*-iodonitrotetrazolium violet (INT) to its coloured product INTH (Babson and Phillips 1965) detectable in a plate reader. Cells were treated with test compounds for 24 h and at the end of the treatment, supernatant medium was collected. Ten microliters of supernatant medium was added to 90 μL of reagent mix (80% TRAM buffer, 20% colour reagent, 3.3 mM β-NAD (Merck, N7004) and 0.33 μL/mL LDH (Merck, L2500)) and incubated in light-protected at room temperature for 5 to 10 min. TRAM buffer contains 108 mM Triethanolamine HCl (Sigma, T9534), 10.7 mM EDTA-Na_2_ (Merck, E4884), 42 mM MgCl_2_ (Merck, M8266) in ddH20, pH 7.5. Colour reagent contains 1.63 mM PMS (Merck, P9625), 3.95 mM INT (Merck, I8377), 35% ethanol, and 2% Triton X-100 (Merck). Optical density was measured in a plate reader at 490 nm and lactate was quantified against a lactate standard curve (Fluka Chemika, 71718) using a spline fit/LOWESS (cubic spline) in GraphPad Prism.

### Cell death assay in 3D cultured HepG2

At day 21, the culture medium was replaced by fresh medium containing the compounds in the desired concentration. During the single exposure scenario, Hoechst (final concentration 667 ng/μL) was added to the exposure medium and after 24 h the medium was replaced with DMEM/F12 containing PI (final conc. 400 nM). For the repeat exposure scenario, Hoechst was added to the 4th exposure and PI to the 5th exposure. Upon 1 h after PI-incubation for the single exposure scenario or 24 h after the 5th repeated exposure, the Hoechst and PI staining were monitored in 11 z-planes using, respectively, the 408 and 561 nm lasers. The confocal imaging was performed using a Nikon TiE2000 with perfect focus system and XY-stage (Nikon, Amsterdam, The Netherlands). Quantification of the Hoechst and PI signal localization was performed using Nis Elements Analysis software. First, a max projection was created of all z-stacks. The overlapping areas between Hoechst and PI in the projection picture were assessed based on a manually curated intensity threshold. Finally, the fraction of spheroids positive for PI staining was determined.

### Microarray in HepG2 cells

Transcriptomic data of a previous HepG2 study from the de Water lab were used to interrogate potential differences in glycolysis gene expression in 3D cultured cells (Hiemstra et al. 2019). The study compared 2D at day 3 and 3D for 3, 7, 14, 21, and 28 days. The Affymetrix HT Human Genome U133 plus platform was used and the original CEL files are stored at GEO (Number: GSE128763). Here, we pulled out the genes from the Panther glycolysis pathway (http://amp.pharm.mssm.edu/Harmonizome/gene_set/Glycolysis/PANTHER+Pathways). Values are represented as fold changes of HepG2 cells cultured in matrix gel for 3, 7, 14, 21 or 28 days over HepG2 cells cultured on plastic for 3 days.

### Data normalization and statistical analysis

All results are average of 2–3 independent experiments. Each independent experiment is referred to as biological replicate and includes at least two technical replicates. At first, normalization is applied by representing responses as percentage of 0.1% DMSO-treated samples (control) for the following assays: resazurin reduction, lactate production, Rho123, JC-1, and PI staining. For those assays, a second normalization was applied setting as 100% (upper or lower asymptote for inhibition or activation curves, respectively), the average of at least 2 non-effective concentrations (if applicable), to be able to calculate the BMC (Table 2) according to the benchmark concentration concept of in vitro toxicology (Krebs et al. 2020). For assays performed in Seahorse, basal OCR/ECAR responses were normalised as percentage of measurements before treatment injection. Maximal OCR was normalised as percentage of maximal respiration of control samples (0.1% DMSO treated). ECAR was normalised by setting the lower asymptote of the response curve to 0%, corresponding to the 100% ECAR prior to compound injection (basal acidification), and the upper asymptote to 100%, corresponding to the maximal ECAR induction (oligomycin response). Variation in all performed assays was calculated and represented as standard deviation (SD). Curves were fit using the non-liner regression four-parameter Hill model. BMC was calculated using the in vitro toxicology on-line tool provided by the group of Prof. Leist, University of Konstanz (Krebs et al. 2020). Significance levels were calculated comparing treatment responses to assay´s specific control using one way ANOVA followed by a Dunnett’s test, *p < 0.05 (Tables 3, 4). Data analysis was performed using Rstudio (Boston, USA) R 3.6.0 and included the following packages dply (Wickham 2011), tidyr (Wickham 2016), data.table (Dowle et al. 2016), multcomp (Hothorn et al. 2008) and stats.

**Table 2:** IC50s and EC50s summary table of all experiments

Compound	Complex	Viability (log10[M]) glucose settings		Viability (log10[M]) galactose settings		Cell death 1 × 24 h (log10[M])	Cell death 5 × 24 h (log10[M])	MMP(log10[M])		Lactate production (log10[M])	
		Resazurin		Resazurin		PI staining	PI staining				
		RPTEC/TERT1	HepG2	RPTEC/TERT1	HepG2	HepG2 3D	HepG2 3D	RPTEC/TERT1	HepG2	RPTEC/TERT1	HepG2
		IC50	IC50	IC50	IC50	EC50	EC50	EC50	EC50	EC50	EC50
Capsaicin	CI	> − 5.00	> − 5.00	− 5.21	> − 5.00	> − 5.00	> − 5.00	> − 5.00	> − 5.00	> − 5.00	> − 5.00
Deguelin	CI	− 6.33	> − 5.00	− 6.10	− 6.46	− 5.41	− 5.72	− 5.35	− 7.21	− 6.62	− 6.61
Fenazaquin	CI	− 5.37	> − 5.00	NT	NT	NT	NT	> − 5.00	− 6.55	− 6.06	− 6.07
Fenpyroximate	CI	− 6.73	> − 5.00	− 6.65	− 7.28	− 6.52	− 6.56	− 6.36	− 8.05	− 6.69	− 6.88
Pyridaben	CI	− 6.12	> − 5.00	NT	NT	NT	NT	− 5.46	− 8.19	− 6.56	− 6.85
Pyrimidifen	CI	− 6.93	> − 5.00	− 6.88	− 7.29	− 5.80	− 5.82	− 6.57	− 8.43	− 6.79	− 7.01
Rotenone	CI	− 6.77	> − 5.00	− 6.79	− 7.23	− 5.89	− 6.54	− 7.29	− 8.09	− 6.65	− 6.90
Tebufenpyrad	CI	− 6.78	> − 5.00	− 5.94	− 6.15	> − 5.00	− 5.13	− 5.44	− 7.29	− 6.05	− 6.84
Carboxine	CII	> − 5.00	> − 5.00	> − 5.00	> − 5.00	> − 5.00	> − 5.00	> − 5.00	> − 5.00	> − 5.00	− 6.92
Fenfuram	CII	> − 5.00	> − 5.00	NT	NT	NT	NT	> − 5.00	> − 5.00	> − 5.00	> − 5.00
Flutolanil	CII	> − 5.00	> − 5.00	NT	NT	NT	NT	> − 5.00	> − 5.00	> − 5.00	> − 5.00
Mepronil	CII	> − 5.00	> − 5.00	> − 5.00	> − 5.00	> − 5.00	> − 5.00	> − 5.00	> − 5.00	> − 5.00	> − 5.00
Thifluzamide	CII	> − 5.00	> − 5.00	> − 5.00	> − 5.00	> − 5.00	> − 5.00	> − 5.00	− 5.06	> − 5.00	> − 5.00
Antimycin A	CIII	− 6.92	> − 5.00	− 7.15	− 7.53	> − 5.00	− 7.13	− 7.36	− 8.21	− 6.68	− 6.25
Azoxystrobin	CIII	> − 5.00	> − 5.00	> − 5.00	> − 5.00	> − 5.00	> − 5.00	− 5.55	− 5.70	− 5.64	− 5.28
Cyazofamid	CIII	> − 5.00	> − 5.00	> − 5.00	> − 5.00	> − 5.00	> − 5.00	− 5.70	> − 5.00	− 5.42	> − 5.00
Fenamidone	CIII	> − 5.00	> − 5.00	NT	NT	NT	NT	> − 5.00	− 5.50	− 5.83	− 5.36
Kresoxim-methyl	CIII	> − 5.00	> − 5.00	NT	NT	NT	NT	> − 5.00	> − 5.00	> − 5.00	− 6.69
Picoxystrobin	CIII	− 5.47	> − 5.00	− 5.32	− 5.48	> − 5.00	> − 5.00	> − 5.00	− 6.14	− 6.32	− 6.26
Pyraclostrobin	CIII	− 5.49	> − 5.00	− 5.85	− 6.04	> − 5.00	> − 5.00	− 5.75	− 6.31	− 6.42	− 6.32
Trifloxystrobin	CIII	> − 5.00	> − 5.00	NT	NT	NT	NT	> − 5.00	> − 5.00	− 5.54	> − 5.00

Compound	ECAR basal (log10[M]) intact cells		OCR basal (log10[M]) intact cells		OCR maximal (log10[M]) intact cells		OCR basal (log10[M]) permeabilized cells		
	RPTEC/TERT1	HepG2	RPTEC/TERT1	HepG2	RPTEC/TERT1	HepG2	HepG2 (IC50)		
	EC50	EC50	IC50	IC50	IC50	IC50	CI	CII/CIII	CIV
Capsaicin	> − 5.00	− 5.04	> − 5.00	> − 5.00	> − 5.00	> − 5.00	− 3.59	NR	NR
Deguelin	− 6.71	− 6.45	− 6.44	− 6.17	− 7.02	− 6.77	− 6.98	NR	NR
Fenazaquin	− 6.05	− 5.62	− 5.84	− 5.59	− 6.44	− 6.42	− 6.74	NR	NR
Fenpyroximate	− 5.90	− 6.28	− 6.27	− 6.17	− 5.93	− 6.53	− 7.72	NR	NR
Pyridaben	− 5.81	− 5.31	− 5.91	− 5.49	− 6.40	− 5.82	− 7.37	NR	NR
Pyrimidifen	− 7.18	− 7.29	− 6.22	− 7.10	− 7.48	− 7.61	− 8.15	NR	NR
Rotenone	− 6.76	− 6.67	− 6.49	− 6.62	− 6.99	− 7.22	− 7.48	NR	NR
Tebufenpyrad	− 6.15	− 6.49	− 5.79	− 6.07	− 6.61	− 6.68	− 5.26	> − 5.00	NR
Carboxine	> − 5.00	> − 5.00	> − 5.00	> − 5.00	> − 5.00	> − 5.00	> − 4.30	− 4.63	NR
Fenfuram	> − 5.00	> − 5.00	> − 5.00	> − 5.00	> − 5.00	> − 5.00	> − 3.30	> − 3.30	NR
Flutolanil	> − 5.00	> − 5.00	> − 5.00	> − 5.00	> − 5.00	> − 5.00	NR	− 4.16	> − 3.30
Mepronil	> − 5.00	> − 5.00	> − 5.00	> − 5.00	> − 5.00	> − 5.00	> − 3.30	− 3.75	NR
Thifluzamide	> − 5.00	> − 5.00	> − 5.00	> − 5.00	> − 5.00	> − 5.00	NR	− 4.71	NR
Antimycin A	− 7.46	− 7.55	− 7.44	− 7.55	− 7.62	− 7.69	− 7.58	− 7.72	NR
Azoxystrobin	− 5.14	> − 5.00	− 5.04	> − 5.00	− 5.20	> − 5.00	− 4.50	− 4.77	> − 4.00
Cyazofamid	− 5.88	> − 5.00	> − 5.00	> − 5.00	> − 5.00	> − 5.00	NR	NR	NR
Fenamidone	− 5.37	− 5.08	− 5.09	− 5.14	− 5.35	− 5.21	− 4.16	− 4.71	NR
Kresoxim-methyl	− 5.28	> − 5.00	− 5.01	− 5.04	− 5.02	> − 5.00	− 5.25	− 5.21	NR
Picoxystrobin	− 5.88	− 5.04	− 5.35	− 5.63	− 5.77	− 5.30	− 5.16	− 5.40	NR
Pyraclostrobin	− 6.12	− 5.93	− 5.90	− 5.86	− 6.22	− 5.76	− 6.07	− 6.41	NR
Trifloxystrobin	− 5.42	> − 5.00	− 5.39	> − 5.00	− 5.37	> − 5.00	− 5.24	− 5.27	> − 4.00

Inhibitory and effective derived concentrations relative to all performed experiments. IC50s values were calculated with the in vitro toxicology on-line tool provided by the group of Prof. Leist, University of Konstanz (Krebs et al. 2020), which calculates the curve fit applying a 4-parameter Hill model to the re-normalized datasets. The equation of the Hill model was solved for *f*(*x*) = 50% to determine the IC50s referring to the concentrations at which an inhibitory effect of 50% was observed. For endpoints increasing with treatment, EC50s values were calculated by fitting the curve without using a model but using a point-to-point curve fit of re-normalized datasets. 1000 points were calculated with the *X* values ranging from lower to highest tested concentration. EC50s values were extrapolated from the curve and correspond to the concentrations at which an increased response of 50% was observed

*NT* not tested, *NR* no response

**Table 3:**
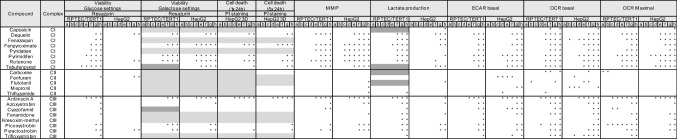
Statistical significance of concentration responses relative to all performed experiments excluding ETC inhibition specificity assay

Significance levels were calculated comparing treatment responses to assay´s specific control using one way ANOVA followed by a Dunnett’s test, *p < 0.05. Light grey = chemical not tested in particular assay, dark grey = not enough replicates to perform statistics. The numbers correspond to the used concentrations in µM: *a* = 0.000128, *b* = 0.0064, *c* = 0.0032, *d* = 0.016, *e* = 0.08, *f* = 0.4, *g* = 7 and *h* = 10

**Table 4:**
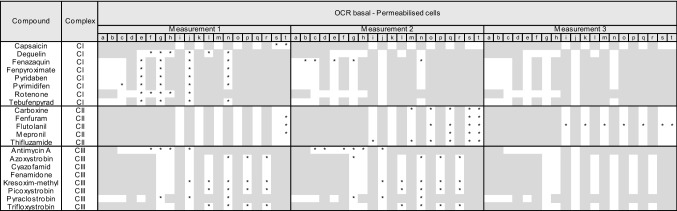
Statistical significance of concentration responses relative to MRC complex inhibition specificity assay

Significance levels were calculated comparing responses to assay controls using one way ANOVA followed by a Dunnett’s test, *p < 0.05. Light grey = chemical not tested in particular assay. The numbers correspond to the used concentrations in µM: *a* = 0.00001, *b* = 0.0001, *c* = 0.001, *d* = 0.00316, *e* = 0.01, *f* = 0.316, *g* = 0.1, *h* = 0.316, *i* = 0.5, *j* = 1, *k* = 1.58, *l* = 3.16, *m* = 5, *n* = 10, *o* = 15.8, *p* = 31.6, *q* = 50, *r* = 100, *s* = 158 and *t* = 500

## Results

### Effects of various selective ETC-complex inhibitors on viability and OCR

**Fig. 2:**
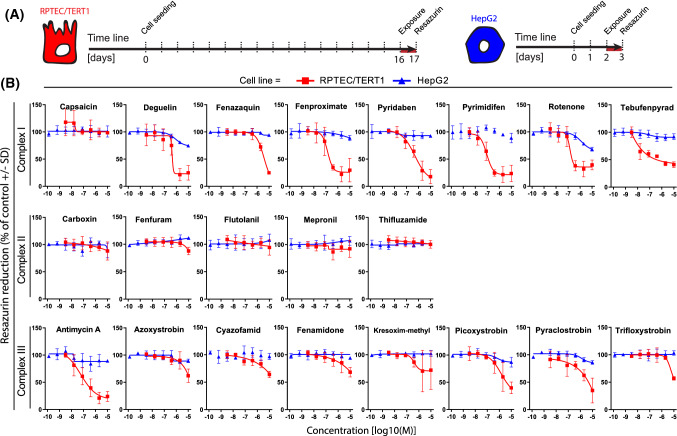
Effect of compound exposure on cellular viability as measured by resazurin reduction. **a** Schematic representation of the experimental setup in RPTEC/TERT1 and HepG2 cells, the red line represents the exposure time. **b** Concentration response curves of resazurin reduction in RPTEC/TERT1 and HepG2 cells exposed for 24 h to a range of concentrations (1.28E−10, 6.40E−10, 3.20E−9, 1.60E−8, 8.00E−8, 4.00E−7, 2.00E−6, 1.00E−5M) of complex I, complex II and complex III inhibitors of the ETC. RPTEC/TERT1 (red) and HepG2 (blue). Values are represented as percentage of vehicle controls (0.1% DMSO) and further normalized to the average of at least two non-effective concentrations (if applicable) set as 100%. Measurements are average of at least three independent experiments ± SD. Connecting lines are non-linear fits (*Y* = bottom + (top − bottom)/(1 + 10^((LogIC50-X) × HillSlope))) (color figure online)

In both cell lines, mitochondrial and metabolic parameters were measured upon exposure to a broad concentration range of in total 21 mitochondrial ETC CI, CII and CIII inhibitors. The capacity of cells to reduce resazurin is widely used as a viability assay due to its ease of use and low cost (Jennings et al. 2004, 2007). Resazurin reduction was measured in both cell types after 24 h exposure of test compounds at a range of concentrations up to 10 µM. Cell viability decreased in a concentration-dependent manner upon exposure to 15 out of 21 complex inhibitors in the RPTEC/TERT1 cell line, whereas only rotenone mildly affected the viability of HepG2 cells (Fig. 2). The CII inhibitors and capsaicin did not affect resazurin reduction up to 10 µM in a 24 h exposure.

**Fig. 3:**
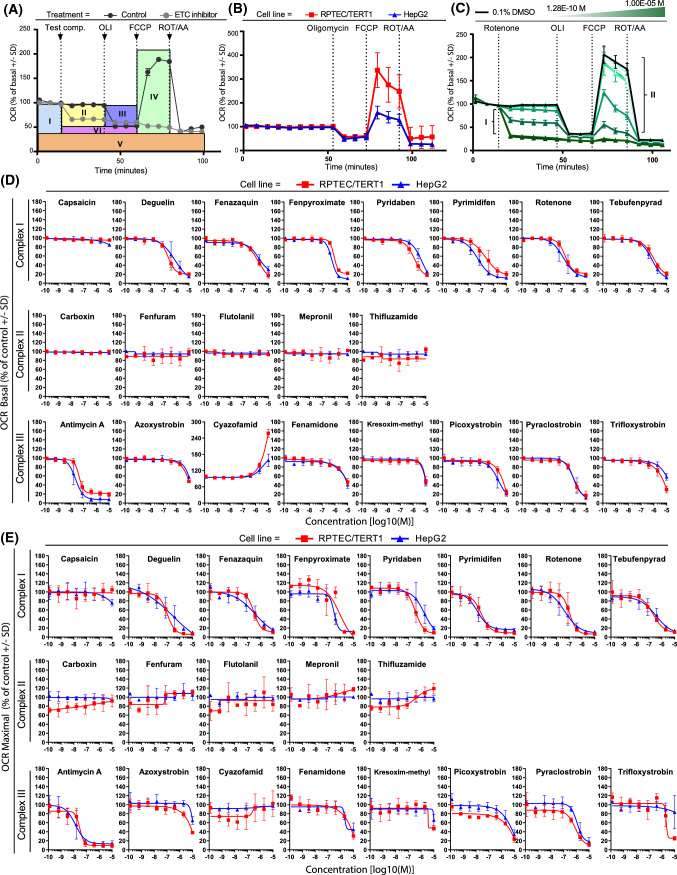

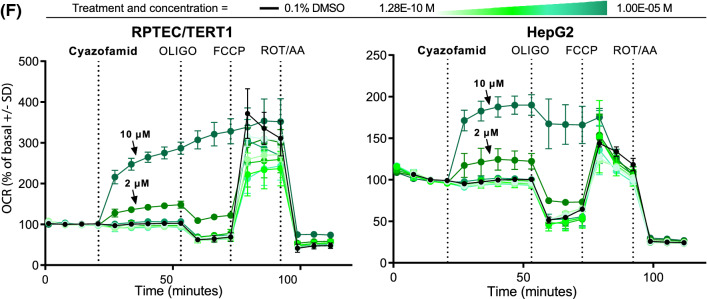
Oxygen consumption rates in untreated and treated RPTEC/TERT1 and HepG2 cells. Effect on key parameters of mitochondrial function measured as changes in OCR with the Seahorse analyser upon 30 min exposure to range of concentrations (1.28E−10, 6.40E−10, 3.20E−9, 1.60E−8, 8.00E−8, 4.00E−7, 2.00E−6, 1.00E−5M) of complex I, complex II and complex III inhibitors of the ETC in RPTEC/TERT1 and HepG2 cells. **a** Overview of measurable parameters after subsequent injections of test compound and modulators of the oxidative phosphorylation of the mitostress assay in HepG2 cells. Respiration is first measured at the basal level of test system (I). Decrease in OCR upon test compound injection, indicate inhibition of the mitochondrial respiration (II). Changes in OCR upon oligomycin addition, indicate the portion of oxygen employed in ATP production (III). OCR increases after the protonophore addition indicates the maximal ability of the cell to increase mitochondrial respiration (IV). Addition of antimycin A and rotenone allows for identification of non-mitochondrial respiration (V). The difference between oligomycin and rotenone/antimycin response indicates the remaining basal respiration not coupled with ATP production to be attributed to proton leakage (VI). Arrows indicate time of injections. **b** OCR changes after mitostress test conducted in 0.1% DMSO control samples in RPTEC/TERT1 and HepG2. Data are represented as mean of at least seven independent experiments, expressed as percentage of basal respiration ± SD. **c** Representative response upon exposure to rotenone (1.28E−10, 6.40E−10, 3.20E−9, 1.60E−8, 8.00E−8, 4.00E−7, 2.00E−6, 1.00E−5M) in HepG2 cells showing a dose dependent effect in basal (I) and maximal (II) respiration rates. **d, e** Plots of concentration responses in terms of oxygen consumption rates extrapolated from the mitostress test of panel compounds. Data represents the mean of two independent experiments ± SD. All measurements were normalized for basal respiration prior to compound injection, slopes are generated by plotting dose responses of the direct oxygen consumption inhibition (OCR basal, D) and inhibition of the uncoupler stimulated respiration (OCR maximal respiration, E), the latter further represented as percentage of untreated controls samples. **f** Response of the mitostress assay after treatment with different concentrations of cyazofamid (1.28E−10, 6.40E−10, 3.20E−9, 1.60E−8, 8.00E−8, 4.00E−7, 2.00E−6, 1.00E−5M). The two highest concentrations indicate the uncoupling effect of the compound in the two cell systems

Mitochondrial oxygen consumption rate (OCR) was quantified in intact RPTEC/TERT1 and HepG2 cells using the Seahorse XFe96 Bioanalyzer (Agilent). OCR was quantified for 30 min immediately after test compound injection to estimate the effect of compound on basal respiration. After 30 min, oligomycin was injected to estimate mitochondrial ATP production. Thereafter, FCCP was injected to provide maximal mitochondrial respiration. Finally, rotenone and antimycin A were injected to assess non-mitochondrial respiration (Fig. 3). Comparison of untreated cells revealed a 2.2-fold higher maximal respiration rates in RPTEC/TERT1 cells compared to HepG2 cells (RPTEC/TERT1 337.4% basal OCR ± 72.9, HepG2 159.4% basal OCR ± 26.8) (Fig. 3b).

CI inhibitors, except for capsaicin, induced a concentration-dependent decrease in basal and maximal OCR in both cell lines (Fig. 3d, e). The effects were comparable in both cell types with pyrimidifen being the most potent of the entire CI class. None of the CII inhibitors exhibited an effect in basal OCR. However, some of the compounds, including thifluzamide, showed a tendency to increase maximal respiration at higher concentrations in RPTEC/TERT1 cells (Fig. 3d, e). CIII inhibitors exhibited a dose-dependent decrease in basal and maximal OCR, in both cell types with the exception of cyazofamid (Fig. 3d, e). Antimycin A was the most potent of the CIII class. Cyazofamid demonstrated a strong uncoupling effect, with a more pronounced effect in RPTEC/TERT1 cells (Fig. 3d). Note that the Y-axis in Fig. 3d is extended to 300% due to this effect, where all other graphs have a scale from 0 to 180%. The Seahorse mitostress OCR plots for cyazofamid are provided in Fig. 3f, which more clearly shows the uncoupling effect at 2 and 10 µM in both cell types.

### Mitochondrial ETC-complex inhibition specificity

**Fig. 4:**
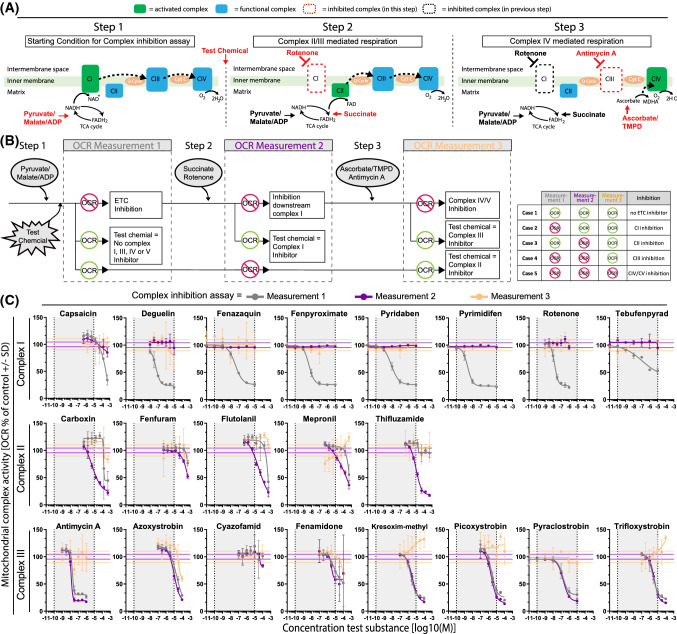
Identification of ETC target using the mitochondrial complex assay. **a** Schematic representation of the ETC complex inhibition assay. The complex inhibition assay consists of a sequential injection (in the same well) of substrates and/or inhibitors to determine specific complex inhibition. Initially cells are treated with permeabilizing agent and CI substrates (pyruvate/malate/ADP) (Step 1). Cells are subsequently injected with test chemical, followed by a second injection with CII substrate (succinate) and CI inhibitor (rotenone) simultaneously (Step 2), and finally a complex IV substrate (ascorbate/TMPD) and CIII inhibitor (antimycin A) is added (Step 3), followed by ORC measurement. **b** Schematic representation of expected OCR responses upon test compound and sequential assay substrates and/or inhibitors addition. Following test chemical injection, OCR is measured (measurement 1, grey line). Decreased OCR indicates an inhibition of the ETC (unknown complex), no effect on OCR indicates either inhibition of CII, case 3 (established in the next assay measurements) or no ETC inhibition (case 1). Addition of rotenone and succinate at step 2, blocks CI and drives CII respectively. OCR is measured after step 2 (measurement 2, purple line). A rescue in decreased OCR indicates the test compound as CI inhibitor (case 2), a continuation in the drop of OCR indicates the site of inhibition is downstream of CI and a decrease in OCR where not observed previously indicates CII inhibition (case 3). Addition of antimycin A and ascorbate/TMPD at step 3, blocks CIII and drives CIV respectively. OCR is measured after step 3 (measurement 3, orange line). A rescue in decreased OCR indicates the test compound as CIII inhibitor (case 4) or confirms the test compound as CII inhibitor (case 3), a continuation in the drop of OCR indicates CIV or CV as the site of inhibition (case 5). **c** Plots of dose responses in OCR for panel compounds in HepG2 cells. OCR is expressed as percentage of baseline response prior to compound exposure, data is mean of 3 independent experiments ± SD. A drop in measurement 1 OCR (grey line) alone indicates inhibition of complex I, drop in measurement 2 OCR (purple line) alone indicates CII inhibition and drop in measurement 1 and measurement 2 together indicates CIII inhibition. A drop in measurement 3 OCR (orange line) indicates CIV or CV inhibition and/or off target effects (color figure online)

To confirm the specificity of the various ETC inhibitors and to rule out confounding pharmacokinetic effects, such as lack of transport or metabolism, Seahorse measurements were conducted in permeabilised HepG2 cells with sequential addition of paired ETC-complex substrates and inhibitors (Fig. 4). All CI inhibitors including capsaicin (50 µM and above) exhibited the expected CI-specificity pattern, i.e. inhibition of OCR after injection and recovery with succinate. All CII inhibitors exhibited CII-specificity pattern, i.e. no (or less potent) OCR inhibition after direct injection but OCR inhibition after succinate/rotenone injection. However, only carboxin and thifluzamide exhibited activity at or below 10 µM. CIII inhibition is determined by OCR inhibition after compound injection, no recovery with succinate, but recovery with ascorbate/TMPD. Antimycin A, kresoxim-methyl, picoxystrobin, pyraclostrobin and trifloxystrobin conformed to this pattern, while azoxystrobin and fenamidone were only partially rescued with ascorbate/TMPD. Cyazofamid exhibited a CII inhibition pattern but only at 100 µM.

### Monitoring mitochondrial membrane potential (MMP)

**Fig. 5:**
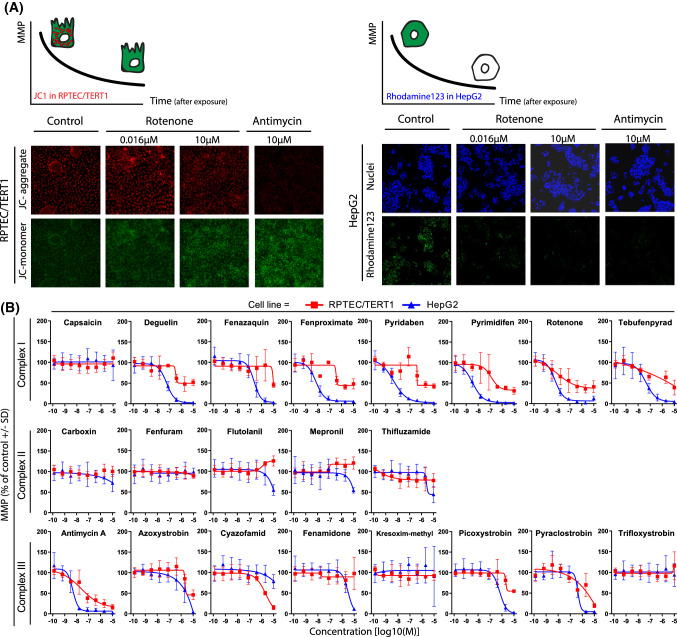
Effect of compound exposure on mitochondrial membrane potential. Effect on mitochondrial membrane polarization by assessment of changes in mitochondrial membrane potential upon 24 h exposure to range of concentrations (1.28E−10, 6.40E−10, 3.20E−9, 1.60E−8, 8.00E−8, 4.00E−7, 2.00E−6, 1.00E−5M) of complex I, complex II and complex III inhibitors of the ETC in RPTEC/TERT1 and HepG2 cells. **a** Schematic representation of mitochondrial membrane depolarization using JC-1 and Rho123 in RPTEC/TERT1 and HepG2 respectively and representative images of changes in mitochondrial membrane polarization in RPTEC/TERT (JC-1) and HepG2 (Rho123) upon exposure to vehicle control, rotenone and antimycin A. **b** Concentration response curves of panel compounds in RPTEC/TERT1 (red) and HepG2 (blue). The Rho123 intensity and the intensity ratio for JC-1 were presented as percentage of 0.1% DMSO exposure. The data was further normalized to the average of at least two non-effective concentrations (if applicable). Measurements are expressed as average of at least three independent experiments ± SD (color figure online)

Live cell imaging was used to measure MMP in HepG2 utilising Rho123, whereas JC-1 dimer/monomer ratio measured with a plate reader was used to assess changes in MMP in RPTEC/TERT1 cells. For reasons, we did not elucidate Rho123 as it was unresponsive in RPTEC/TERT1 cells and, hence, the JC-1 dye was used. A decrease in Rho123 intensity is representative for reduced MMP, while JC-1 red/green ratio shifts to the green with decreasing MMP (Fig. 5a). MMP decreased in a concentration-dependent manner in both cell types with CI inhibitors, except for capsaicin (Fig. 5b). CII inhibitors did not decrease MMP in RPTEC/TERT1 cells; flutolanil and mepronil actually increased it. In HepG2 cells, flutolanil, mepronil and thifluzamide decreased MMP, but only at the highest concentrations. Exposure to all CIII inhibitors, except for kresoxim-methyl and trifloxystrobin, led to decreased MMP in both cell systems.

### Increased glycolysis by inhibitors of CI and CIII

**Fig. 6:**
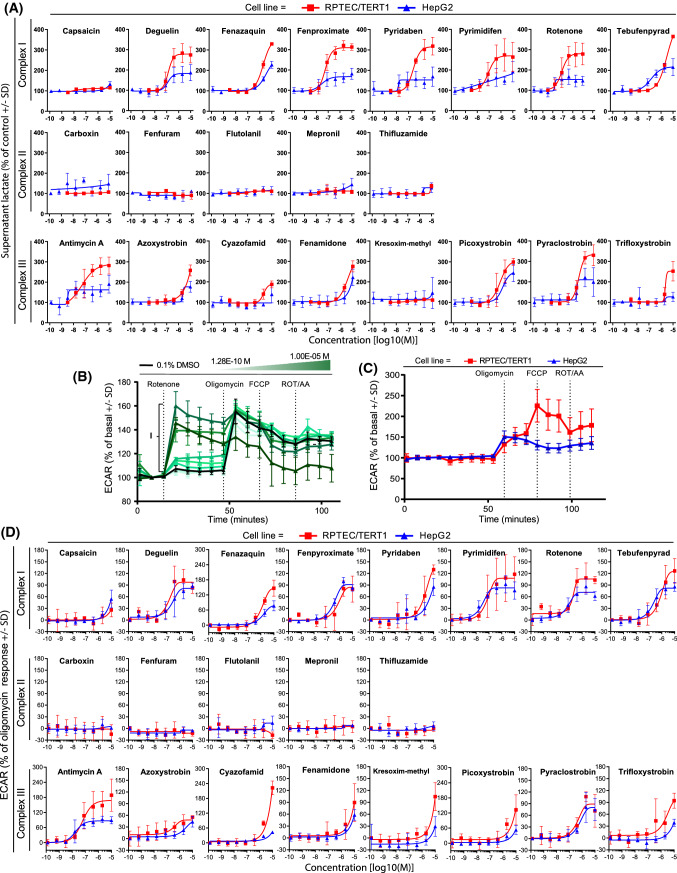
Effect of compound exposure on extracellular lactate and extracellular acidification rates. Glycolytic switch upon decreased mitochondrial respiration was indirectly assessed by measurements of supernatant lactate and the extracellular medium acidification after 24 h exposure to range of concentrations (1.28E−10, 6.40E−10, 3.20E−9, 1.60E−8, 8.00E−8, 4.00E−7, 2.00E−6, 1.00E−5M) of complex I, complex II and complex III inhibitors of the ETC in RPTEC/TERT1 and HepG2 cells. **a** Levels of lactate in the supernatant medium. Data are represented as percentage of 0.1% DMSO controls and re-normalized to the average of at least two non-effective concentrations (if applicable) set as 100%. **b** Representative response to the testing concentration range of rotenone in HepG2 cells showing a dose dependent increase of medium acidification (I) reflecting the glycolytic turnover increase. **c** Changes in ECAR after mitostress test conducted in 0.1% DMSO control samples in RPTEC/TERT1 and HepG2. Data are represented as mean of at least seven independent experiments, expressed as percentage of basal acidification ± SD. **d** Plots of concentration responses of changes in ECAR after panel compounds injection. Data is mean of two independent experiments ± SD. Measurements are expressed as percentage of basal acidification and further normalized by setting the lower asymptote of the response curve to 0%, corresponding to the 100% ECAR prior to compound injection (basal acidification), and the upper asymptote to 100%, corresponding to the maximal ECAR induction (oligomycin response)

Exposure to CI and CIII inhibitors for 24 h resulted in an increase in supernatant lactate in both cell types, with the exception of capsaicin and kresoxim-methyl. No effect was observed when exposing cells to CII inhibitors at tested concentrations (Fig. 6a). Overall, RPTEC/TERT1 cells showed a more pronounced increased glycolysis compared to HepG2 cells. Utilising the Seahorse measurement of ECAR, after direct injection of compounds, a rapid increase in response to CI and CIII inhibitors was observed, with no increase in ECAR for CII inhibitors (Fig. 6b–d). Similar ECAR responses were observed in both cell systems, although cyazofamid was much more potent in RPTEC/TERT1 cells. Both cell types showed a robust increase in ECAR in response to oligomycin; however, only RPTEC/TERT1 further increased ECAR in response to FCCP (Fig. 6c).

### Glu/Gal switch sensitises towards ETC inhibition-induced toxicity in HepG2 cells but not RPTEC/TERT1 cells

**Fig. 7:**
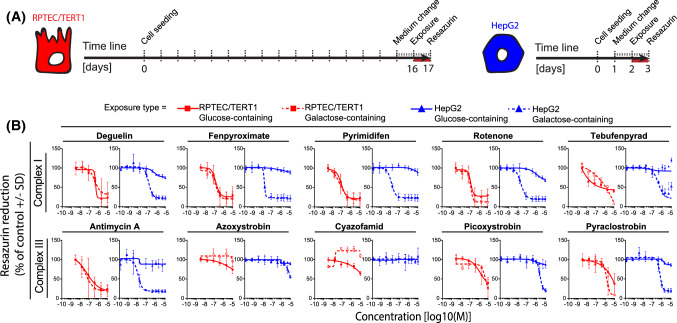
Effect of medium glucose on compound induced alterations in cell viability. The effects of medium switch (glucose to galactose) in terms of cell viability was assessed in RPTEC/TERT1 and in HepG2 after 24 h exposure to a range of concentrations (1.28E−10, 6.40E−10, 3.20E−9, 1.60E−8, 8.00E−8, 4.00E−7, 2.00E−6, 1.00E−5M) of complex I, complex II and complex III inhibitors of the ETC. **a** Schematic representation of the carbon source switch from glucose to galactose-containing medium in RPTEC/TERT1 (5 mM Glu/Gal) and HepG2 (25 mM Glu/Gal) respectively, the red line represents the exposure time. **b** Plots of concentration responses in resazurin reduction. Measurements are expressed as percentage of vehicle controls (0.1% DMSO) and further normalized to the average of at least two non-effective concentrations (if applicable) set as 100%. Values are mean of two to four independent experiments ± SD. Connecting lines are non-linear fits (*Y* = bottom + (top − bottom)/(1 + 10^((LogIC50-X) × HillSlope)))

We evaluated the effect of glucose removal on the sensitivity of RPTEC/TERT1 and HepG2 cells to a restricted compound panel of CI and CIII inhibitors (Fig. 7). Cells were switched from glucose to galactose containing medium one day before chemical exposure (Fig. 7). Cell viability was measured after 24 h exposure to a subset of compounds. Replacing glucose with galactose had no clear effect on the viability to chemical exposure in RPTEC/TERT1 cells (Fig. 7). In contrast, galactose conditions strongly sensitised HepG2 cells to the OCR active compounds, underlining the dependency of HepG2 cells on glycolysis under mitotoxicant-induced stress conditions and making this system more comparable to the RPTEC/TERT1 cells (Fig. 7).

### Effect of 3D spheroid culture and repeated exposures in HepG2 cells

**Fig. 8:**
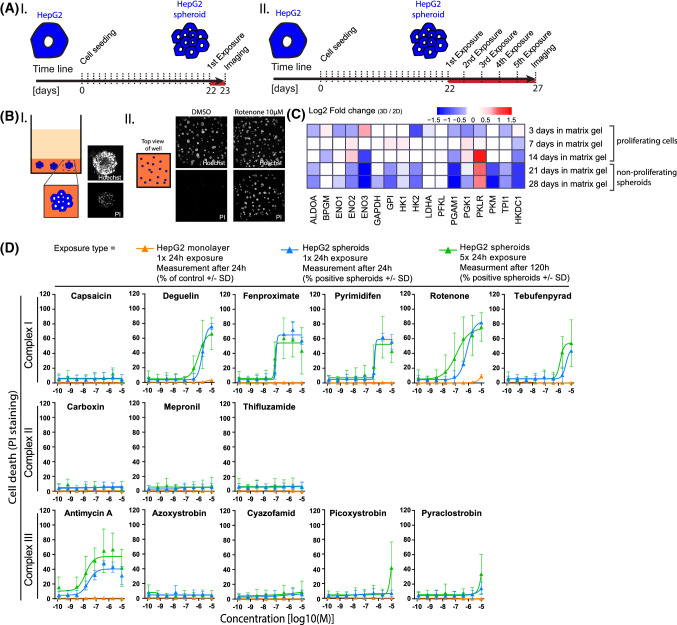
Comparison of compound induced toxicity in 2D cultured HepG2 and 3D HepG2 spheroids. **a** Schematic representation of cell culture/differentiation protocol and time of endpoint measurements in HepG2 spheroids treated with two exposure regimes; I = 1 × 24 h exposure, measured after 24 h and II = 5 × 24 h exposure, measured after 120 h. **b** (I) Schematic representation of HepG2 spheroids in a 384 well, with a representative picture of a single spheroid stained with nuclear marker (Hoechst) and cell death marker (PI). (II) Representative images of a 384 well with spheroids cultured in glucose-containing medium followed by exposure to 10 µM rotenone or DMSO (24 h) and stained with nuclear marker and cell death marker. **c** Heatmap of changes in glycolytic enzyme genes during HepG2 spheroids maturation, in medium containing glucose, showing the evolution toward a less glycolytic state. Log2 fold changes represent the expression of untreated HepG2 cells cultured in matrix gel (3D) at day 3, 7, 14 (proliferating cells) and 21, 28 (non-proliferating spheroids) over untreated HepG2 cells cultured on plastic (2D) for 3 days. **d** Difference in the cytotoxicity responses upon treatment with a range of concentrations (1.28E−10, 6.40E−10, 3.20E−9, 1.60E−8, 8.00E−8, 4.00E−7, 2.00E−6, 1.00E−5M) of CI, CII and CIII inhibitors in HepG2 monolayer (2D) and HepG2 spheroids (3D) with a 1 x 24 h exposure or a consecutive 5 × 24 h exposure. All conditions are in glucose containing medium. Cell death endpoint was assessed with PI staining at the end of treatments. Values are expressed as percentage of PI positive cells/spheroids ± SD and are mean of two or three independent experiments (color figure online)

There is evidence to suggest that HepG2 cells cultured in 3D spheroids results in decreased proliferation and reduced reliance on glucose (Hiemstra et al. 2019). Thus, we investigated if HepG2 3D spheroids had an increased sensitivity to the ETC inhibitors. We compared the effect of 24 h exposure to a range of concentrations of a set of CI, CII and CIII inhibitors in HepG2 monolayer and HepG2 spheroids (Fig. 8d). The combination of the cellular nuclear staining (Hoechst) and the cell death staining (PI) showed a substantial increase in cell death in HepG2 spheroids-treated samples (Fig. 8d, blue curve) when compared to HepG2 monolayer-treated cells (Fig. 8d, orange curve). This effect was more prominent in most of the CI inhibitor-treated samples and antimycin A. In contrast, only visible for the highest concentration of pyraclostrobin was seen in the remaining CIII inhibitor-treated samples. Capsaicin and CII inhibition did not show increased sensitivity in spheroids compared to the 2D model.

Since 3D HepG2 can also be utilised over longer exposure periods, we investigated the effect of a 5 day repeated 24 h exposure (5 × 24 h) compared to the 24 h bolus exposure (1 × 24 h). The 5 × 24 h exposure, increased sensitivity to rotenone (EC50 127 nM 1 × 24 h, 28 nM 5 × 24 h) and antimycin A (EC50 > 10 µM 1 × 2 4 h, 74 nM 5 × 24 h) (Fig. 8d, Table 2).

### Correlation plots

**Fig. 9:**
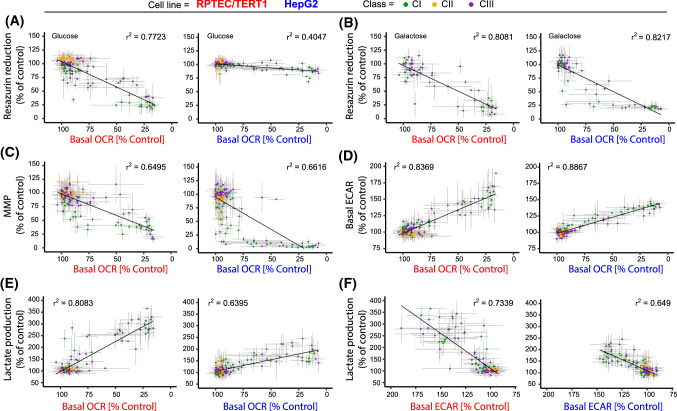
Assay correlation plots. Graphs A to E show the correlation of the highly sensitive 30 min OCR Seahorse measurement with the other assays for all conditions. Each graph relates the OCR response, per compound and per concentration, to the one obtained with the correlating assay with the same treatment condition. Data include the mean of all replicates ± SD, slope’s *r*^2^ values are provided. Classes are distinguished by colour, CI inhibitors (green), CII inhibitors (orange) and CIII inhibitors (purple). **a** Basal 30 min OCR vs 24 h resazurin in glucose settings. **b** Basal 30 min OCR vs 24 h resazurin in galactose settings. **c** Basal 30 min OCR vs 24 h MMP. **d** Basal 30 min OCR vs basal 30 min ECAR. **e** Basal 30 min OCR vs 24 h supernatant lactate. **f** This graph has the same metrics as the other graphs but shows the correlation of 30 min Seahorse ECAR measurement with 24 h supernatant lactate measurement. Note the *X*-axis is reversed for clarity (color figure online)

To give an overview of the data and to explore the relationship between OCR/ECAR and the viability, lactate and MMP assays, correlation plots of all the data were generated (Fig. 9). Matched data for 24 h resazurin reduction, 24 h MMP, 24 h supernatant lactate concentration and 30 min ECAR are plotted vs basal 30 min OCR data (Fig. 9). In addition, 24 h lactate production is plotted vs 30 min basal ECAR (Fig. 9). In RPTEC/TERT1 cells, acute basal OCR correlated with 24 h viability, as measured by resazurin reduction (Fig. 9a, b) (glucose conditions *r*^2^ = 0.7723, galactose conditions *r*^2^ = 0.8081), with 24 h MMP, as measured by JC-1 ratio (Fig. 9c) (*r*^2^ = 0.6495), with acute ECAR (Fig. 9d) (negative correlation, *r*^2^ = 0.8369) and with 24 h supernatant lactate (Fig. 9e) (negative correlation, *r*^2^ = 0.8083). Supernatant 24 h lactate correlated with acute ECAR (Fig. 9f) (*r*^2^ = 0.7339). In HepG2 cells, there was a poor correlation of acute basal OCR with 24 h viability under glucose conditions (Fig. 9a) (*r*^2^ = 0.4047). However, this improved under galactose conditions (Fig. 9b) (*r*^2^ = 0.8217). Acute basal OCR correlated with MMP, as measured by Rho-123 (Fig. 9c) (*r*^2^ = 0.6661), with acute basal ECAR (Fig. 9d) (negative correlation, *r*^2^ = 0.8867) and with 24 h supernatant lactate (Fig. 9d) (negative correlation, *r*^2^ = 0.6395). Supernatant lactate correlated with acute ECAR (Fig. 9c) (*r*^2^ = 0.649), although the distribution range is smaller than in RPTEC/TERT1 cells.

## Discussion

 We have assessed a panel of proposed ETC inhibitors in two human cell lines, with several assays and in various different modes to provide a basis for the establishment of a comprehensive workflow for assessing the impact of chemicals on mitochondrial function and the cellular consequences thereof.

Given the central role of mitochondria in energy metabolism, tissue health and ageing, there is increasing concern regarding the long-term effect that xenobiotics may have on mitochondrial function. Mitochondrial perturbations are likely to increase sensitivity to xenobiotics, decrease cellular repair mechanisms and contribute to both chronic disease states and accelerated ageing (Will et al. 2019). The most direct method to assess mitochondrial activity is by measuring oxygen consumption rates (OCR). Situations that impair mitochondrial function can lead to a decrease in cell viability, but usually there is first an increase in glycolytic rates. Altered glycolytic status can be quantified by assessing extracellular acidification rates (ECAR), or biochemically by measuring supernatant lactate (Limonciel et al. 2011). The Seahorse bioanalyser coupled with the mitostress assay is becoming an industry standard for the quantification of OCR and ECAR (Divakaruni et al. 2014; Eakins et al. 2016; Tilmant et al. 2018). Other methods to assess mitochondrial function include live cell dyes, which under optimised conditions can be related to MMP or comparing toxicity in the presence and absence of glucose as an energy source.

Respiration rates were similarly affected in both RPTEC/TERT1 and HepG2 cells exposed for 30 min to the 21 compounds. In sharp contrast, the effect of ETC inhibition on cell viability after 24 h exposure was cell type- and test system-dependent. Differentiated RPTEC/TERT1 cell viability correlated with OCR inhibition independently of the presence of glucose. HepG2 cell viability correlated with OCR inhibition only in the absence of glucose. Interestingly, culturing HepG2 as 3D spheroids sensitised the cells to OCR inhibition in the presence of glucose. Previous studies have demonstrated that 3D spheroid culture of HepG2 cells decreases proliferation, increases differentiation and increases sensitivity to various compounds with human drug-induced liver injury liability (Ramaiahgari et al. 2014; Hiemstra et al. 2019). Taken together, the data suggest that proliferating cells are less reliant on oxidative phosphorylation as an energy source where glucose is not limiting and that 3D HepG2 cells switch towards an oxidative phosphorylation-mediated energy source.

With the exception of capsaicin, all of the CI inhibitors acutely reduced OCR and enhanced ECAR. In the mitochondrial complex assay, decreased OCR could be rescued with the addition of succinate confirming CI as the site of inhibition for these compounds. Pyrimidifen, deguelin and rotenone were the most potent CI compounds with IC50s in the nanomolar range. Capsaicin was also confirmed as a CI inhibitor, albeit with lower potency (LOEL 50 µM and IC50 approximately 260 µM).

CII inhibitors did not alter cell viability, basal OCR, ECAR or lactate production in living, non-permeabilised cells at concentrations up to 10 µM. However, in the permeabilised assay, all compounds were confirmed as CII specific, albeit at concentrations, close to or above, the maximum concentration tested in intact cells. This difference can be attributed to the fact that CII activity is less critical for electron transfer and ATP production than CI and CIII and is neither required for CI to CIII electron transfer, nor does it participate in proton pumping. CII gives a minor contribution to the Q-cycle compared to CI and, therefore, its inhibition can be totally (thifluzamide) or partially (carboxin, fenfuram, flutolanil and mepronil) masked from CI activity, depending on the strength of the CII inhibition. Indeed, it has been demonstrated that CII activity is more important for ATP generation where energy demand is high (Pfleger et al. 2015) or when CI substrates are limiting (Salabei et al. 2014). However, since CII is directly coupled to the Kreb’s cycle, it would be expected that CII inhibition would eventually negatively impact OCR due to NADH and FADH_2_ depletion, affecting CI and CII activity, respectively. Also, under our experimental settings, pyruvate was present, which may be enough to supply the Krebs cycle until the succinate oxidation step, thus, limiting depletion of NADH. Flutolanil and mepronil do appear to slightly increase MMP in RPTEC/TERT1 cells, although this was not significant. There was also a non-significant tendency of CII compounds to increase maximal OCR in RPTEC/TERT1 cells. These effects are possibly a compensatory mechanism of CII inhibition leading to inner mitochondrial hyperpolarisation. However, further investigations would need to be conducted to address this possible mechanism specifically.

The mitochondrial complex assay confirmed CIII as the site of inhibition of 5 of the 8 compounds previously classified as CIII inhibitors. Contrary to how the system senses the electron flow from CII, CIII inhibition results in the total block of the Q-cycle as it receives electrons from both CI and CII; therefore, concomitant inhibition of CI and succinate addition does not further decrease OCR when CIII inhibitors are applied. Azoxystrobin and fenamidone were only partially rescued by ascorbate/TMPD, which may indicate off-target activity at CIV/CV or some other non-specific activity. Antimycin A was the most potent CIII inhibitor, with IC50s for most parameters in the nanomolar range. Since CIII receives electrons from both CI and CII, complete CIII inhibition would be expected to have a major impact, as it is the case for antimycin A. It appears we did not achieve full CIII inhibition for azoxystrobin, fenamidone, trifloxystrobin or kresoxim-methyl as OCR inhibition did not reach 100%. Surprisingly, cyazofamid did not inhibit OCR, on the contrary, it was the only test compound that demonstrated potent uncoupling effects, evidenced by an increase in basal OCR in the mitostress assay. Thus, under our assay settings, cyazofamid is a potent mitochondrial uncoupler and is, therefore, unlikely to be a classical CIII inhibitor as previously described (Li et al. 2014). While cyazofamid is small and lipophilic, it does not conform to the primary characteristic of classical protonophoretic compounds, such as FCCP and PCP, which are weak acids. This feature is necessary to facilitate the transfer a hydrogen from the inner membrane space to the mitochondrial matrix (Benz and McLaughlin 1983). Thus, the protonophoretic effect of cyazofamid is potentially atypical and requires further attention to identify whether CIII is involved.

Risk assessment is intrinsically linked to exposure duration and frequency of the exposure. To assess the effects of repeated exposures to ETC inhibitors including possible sensitisation towards cellular toxicity, we exposed 3D HepG2 spheroids for 5 consecutive days to 24 h administration to a selection of 14 of the ETC inhibitors and compared the results to a one time 24 h exposure. Repeated exposure increased the toxic sensitivity for rotenone, tebufenpyrad, antimycin A, picoxystrobin and pyraclostrobin, but not to the other compounds tested. However, the increased sensitivity was minimal and does not fully justify the extra effort involved. This may be explained by the fact this set of compounds are very fast acting (within 5 min) and are, thus, unlikely to have accumulative effects of ETC inhibition upon sequential exposures. Also, it is possible that unless there is a major inhibition of the ETC, compensatory mechanisms, such as increased glucose utilisation, can cover the energy deficit. We caution that this should not be assumed for other chemicals, in particular when mitochondrial toxicity is caused through indirect mechanisms, such as inhibition of mitochondrial DNA replication (Dykens and Will 2007; Nadanaciva et al. 2007).

A major current emphasis in transitioning mode of action toxicology to risk-assessment regimes is the focus on adverse outcome pathways, including identification of molecular initiating events (MIE) and key events (KE), leading to a particular pathology (Leist et al. 2017). The current data support a generic AOP framework for mitochondrial ETC inhibition, where CI and CIII inhibition directly leads to a decreased mitochondrial respiration. This leads almost immediately to an increase in glycolysis, which can accomplish energetic needs in cells less reliant on oxidative phosphorylation and where glucose is not limiting. Thus, CI or CIII inhibition is the MIE and decreased OCR is the initial KE and increased ECAR and/or increased supernatant lactate is the second KE. Decrease in MMP might be indicative of OCR inhibition although the correlations with OCR were not very strong. The Seahorse bioanalyser is an ideal method for the proposed AOP as it can simultaneously measure both KEs in real time. However, this becomes more cumbersome at longer exposures or repeated exposures, where supernatant lactate and cellular viability could be used to fill data gaps.

In summary, the study demonstrates the utility of two commonly used cell lines, together with OCR, ECAR, MMP, supernatant lactate and viability to establish critical values to assess chemical-induced ETC inhibition. HepG2 cells gave similar patterns with respect to OCR inhibition as differentiated RPTEC/TERT1; however, glucose-free conditions or 3D spheroid cultures were required to cause ETC inhibition-induced cytotoxicity in HepG2 cells. For studies investigating mitochondrial effects of compounds, we highly recommend the use of cell types and/or experimental conditions that favour oxidative metabolism over glycolysis. This is of particular importance when little is known about the test compound and more complicated mitochondrial perturbations than direct ETC inhibition are possible. Overall, the study presents a comprehensive example of a mitochondrial assessment workflow and establishes measurable key events of CI and CIII ETC inhibition.
